# Supplementary material for: Genetic Control of Photosynthesis in Sugarcane During Successive Ratoon Cycles
Source: Biology (Basel). 2025 Dec 31;15(1):75. doi: 10.3390/biology15010075 (PMC12784760; doi:10.3390/biology15010075)
Supplement: Supplementary file 1 [file biology-15-00075-s001.zip › biology-3992798-supplementary.pdf]

Table S1. Information on the parentages of 74 sugarcane genotypes.

NO.	Genotype	place of origin	NO.	Genotype	place of origin
1	Co1001	India	38	GT08-297	Guangxi
2	CP00-110	America	39	GT09-03	Guangxi
3	CP01-1372	America	40	GT11	Guangxi
4	CP06-2042	America	41	GT11-1076	Guangxi
5	CP72-1210	America	42	GT13-386	Guangxi
6	CP81-1254	America	43	GT42	Guangxi
7	CP85-1308	America	44	GT94-119	Guangxi
8	CP88-1762	America	45	GT96-211	Guangxi
9	CP89-2143	America	46	GUC41	America
10	CP94-1100	America	47	GUC45	America
11	CP94-1340	America	48	GUC46	America
12	CP96-1257	America	49	HoCP91-555	America
13	CP96-1602	America	50	HoCP95-988	America
14	CP97-2730	America	51	HZ28	Hainan
15	CT89-103	Sichuan	52	LC03-182	Guangxi
16	FN04-1027	Fujian	53	LC09-15	Guangxi
17	FN07-2020	Fujian	54	LC09-19	Guangxi
18	FN09-6201	Fujian	55	MT11-610	Fujian
19	FN10-0574	Fujian	56	MT12-1404	Fujian
20	FN11-2907	Fujian	57	Q202	Australia
21	FN40	Fujian	58	ROC1	Taiwan
22	FN94-0744	Fujian	59	ROC11	Taiwan
23	FR99-49	Fujian	60	ROC22	Taiwan
24	GT02-1156	Guangxi	61	ROC25	Taiwan
25	GT02-351	Guangxi	62	ROC26	Taiwan
26	GT02-390	Guangxi	63	YG16	Guangdong
27	GT02-467	Guangxi	64	YG24	Guangdong
28	GT02-619	Guangxi	65	YT89-240	Guangdong
29	GT02-761	Guangxi	66	YT91-976	Guangdong
30	GT03-2309	Guangxi	67	YT94-128	Guangdong
31	GT04-1007	Guangxi	68	YT96-86	Guangdong
32	GT04-1045	Guangxi	69	YZ11-1074	Yunnan
33	GT05-322	Guangxi	70	YZ89-159	Yunnan
34	GT05-375	Guangxi	71	YZ89-7	Yunnan
35	GT05-3846	Guangxi	72	YZ99-596	Yunnan
36	GT07-713	Guangxi	73	ZT1202	Hainan
37	GT08-278	Guangxi	74	ZZ1	Guangxi

Table S2. Principal component analysis for photosynthetic efficiency and factor weight of sugarcane.

Traits	PC1	PC2	PC3
**Fm**	-0.158	**0.987**	-0.027
**Fo**	0.214	**0.977**	0.011
**Fv**	0.318	**0.947**	0.023
**Fv/Fm**	**0.987**	-0.113	0.110
**Fv/Fo**	**0.984**	-0.122	0.105
**Y(NO)**	**-0.987**	0.113	-0.110
**SPAD**	-0.358	0.034	**0.933**
**Eigenvalue**	3.218	2.866	0.907
**Proportion of Variance**	45.97	40.95	12.96
**Cumulative Proportion**	45.97	86.92	99.88
**SS (%)**	46.03	41.00	12.98
